# Acute effects of aerobic versus anaerobic running on heart rate variability and autonomic nervous system indices in male college athletes: a within-subject comparison

**DOI:** 10.3389/fphys.2026.1801021

**Published:** 2026-06-30

**Authors:** Mine Taskin, Maya Budak, Ibrahim Halil Sahin, Nazli Deniz Ceridhan, Halil Taskin

**Affiliations:** 1 Ali Akkanat School of Applied Sciences, Selçuk University, Beyşehir-Konya, Türkiye; 2 Ministry of National Education, Fatih Vocational and Technical Anatolian High School, Erzincan, Türkiye; 3 Faculty of Sport Sciences, Esenyurt University, Istanbul, Türkiye; 4 Faculty of Sport Sciences, Selçuk University, Konya, Türkiye

**Keywords:** aerobic and anaerobic exercise, autonomic nervous system, college athletes, heart rate variability, psychophysiological stress

## Abstract

**Background:**

During rhythmic exercise, overall, heart rate variability (HRV) decreases with increasing intensity, and resting measurements do not reliably reflect responses during activity. Nonneural oscillations in the high-frequency band caused by increased respiratory effort further complicate HRV interpretation. Autonomic nervous system (ANS) balance, particularly between parasympathetic and sympathetic activity, is crucial for optimal athletic performance, highlighting the importance of non-invasive assessments. This study aimed to examine the acute effects of aerobic versus anaerobic running on HRV and ANS indices in male college athletes.

**Methods:**

We evaluated the effects of rest, aerobic running (AeR) and anaerobic running (AnR), recovery on HRV and ANS indices in 25 healthy male college athletes. Each athlete ran for a mean of 30.96 minutes at low intensity for aerobic running, and an average of 2.59 minutes at high intensity for anaerobic running. RR intervals were recorded using a Polar watch with H10 chest strap and analyzed with Kubios HRV Scientific software using time- and frequency-domain HRV measures, including low-frequency (LF, 0.045–0.15 Hz) and high-frequency (HF, 0.15–1.0 Hz) components.

**Results:**

Significant differences were found between AeR and AnR in terms of LF, HF, SNS index, and LF/HF ratio. Differences were also found in RMSSD, SD1, SD2, SNS index, and pNN50 values ​​after AeR and AnR (P<0.05). In within-subject comparisons, during AeR, RMSSD, SD2, pNN50, and PNS index decreased, but SD1 and SNS increased significantly (P<0.05). RMSSD and pNN50 showed no increase or decrease after AeR. SD1 and SNS index decreased after AeR on the other hand, PNS index increase (P<0.05). During AnR, RMSSD, SD2, pNN50, LF, LF/HF ratio decreased, but HF, SD1 and SNS index increased significantly (P<0.05). Compared to the resting state, SD1 and SD2 did not show any change after AnR, but LF, LF/HF ratio, SD1, and SNS index increased, while RMSSD, pNN50, HF, and PNS index decreased.

**Conclusion:**

It was found that the SNS was more dominant during both AeR and AnR. The SNS was more activated during AnR than during AeR. It was observed that college athletes were exposed to more psychophysiological stress during AnR. During the 10-minute post-run recovery period, PNS activation approaches pre-run levels, while SNS activation tends to be higher. The body needs more than 10 minutes to recover after an AnR.

## Introduction

Heart rate variability (HRV) represents the physiological variation in the time interval between consecutive heartbeats, the net effect of multiple regulatory inputs, including autonomic, respiratory, and cardiovascular influences on the sinoatrial node (Banerjee, 2026). Also, it provides a non-invasive window into the heart’s capacity to respond to regulatory inputs mediated by the autonomic nervous system (ANS) (Berntson and Cacioppo, 2004). The measurement quantifies the beat-to-beat alterations in heart rate, primarily mediated by the dynamic balance between sympathetic nervous system (SNS) and parasympathetic nervous system (PNS) activity. Due to its non-invasive nature, ease of measurement, and the breadth of physiological information it provides, HRV has been widely utilized in affective science, psychology, and exercise science to assess autonomic regulation, cardiovascular health, and physiological adaptability (Shaffer and Ginsberg, 2017).

The ANS consists of two primary branches: the SNS and the PNS, both of which play crucial roles in maintaining physiological homeostasis. HRV is widely accepted as an indicator of the functional integrity and interaction of these two branches (Freeman et al., 2006; Boudehri et al., 2023). Generally, higher HRV is associated with efficient autonomic regulation and greater physiological resilience, whereas lower HRV is linked to autonomic dysfunction, increased psychophysiological stress, and potential overtraining (Lundstrom et al., 2023). The interaction between SNS and PNS activity is highly dynamic; for instance, during stress conditions, parasympathetic withdrawal and sympathetic activation lead to characteristic reductions in HRV (Callara et al., 2021).

In the frequency domain, high-frequency (HF) power is generally accepted as a marker of vagal modulation, whereas low-frequency (LF) power and the LF/HF ratio reflect a more complex interaction between sympathetic and parasympathetic influences (Mendonca et al., 2009). The proper functioning of the ANS is essential for both health and athletic performance (Daniela et al., 2022). Maintaining an optimal balance between sympathetic and parasympathetic activity is critical for performance, recovery, and adaptation (Lo et al., 2008; Lamberts et al., 2010). Acute exercise typically results in a decrease in parasympathetic indices such as RMSSD, while sympathetic-related markers may show modest increases (Chiang et al., 2024). These responses reflect the immediate autonomic adjustments required to meet metabolic demands.

The magnitude and direction of HRV responses are influenced by exercise intensity, duration, and modality. High-intensity exercise does not always lead to improved vagal-related HRV, as trained individuals may sometimes exhibit values comparable to sedentary individuals under specific conditions (Buchheit et al., 2004, 2005). In contrast, regular low-to-moderate intensity exercise has been shown to attenuate age-related declines in autonomic function (Uusitalo et al., 2002, 2004). Long-term exercise interventions may improve ANS balance, often reflected by reductions in the LF/HF ratio; however, these adaptations depend on individual characteristics and training variables (Zhang et al., 2025).

Different exercise modalities also produce distinct autonomic responses. For example, resistance training appears to have minimal effects on resting HRV in healthy young adults, although it may enhance parasympathetic modulation in middle-aged individuals with autonomic dysfunction. Nevertheless, acute resistance exercise consistently reduces parasympathetic activity regardless of age (Kingsley and Figueroa, 2016). Similarly, studies in clinical populations, such as patients with coronary artery disease, have reported significant changes in LF and LF/HF indices following exercise training, while HF power remains relatively unchanged (Manresa-Rocamora et al., 2021).

HRV is widely used to assess ANS activity; however, it is well-recognized that HRV signals during dynamic exercise are inherently non-stationary, which can limit the interpretability of traditional frequency-domain metrics such as LF and HF power (Gronwald and Hoos, 2020; Brockmann and Hunt, 2023). Brockmann and Hunt (2023) reported that HRV generally decreases over the course of exercise and with increasing exercise intensity, with intensity-dependent reductions being more pronounced and physiologically significant than time-dependent changes, emphasizing the critical influence of exercise intensity on autonomic regulation.

Despite the extensive use of HRV in exercise science, inconsistencies in literature persist. Some studies report no significant autonomic adaptations following training, which may be attributed to insufficient exercise intensity or volume (Hedelin et al., 2000). A previous study investigating the acute effects of aerobic exercise on autonomic cardiac control has reported significant alterations in HRV parameters. Specifically, improvements have been observed in time-domain parameters such as the root mean square of successive RR interval differences (rMSSD), a marker of parasympathetic activity, within the first hour and up to 24 hours following a single exercise session. In addition, frequency-domain measures, particularly HF power, have also been shown to increase in the early recovery period, reflecting enhanced parasympathetic modulation (Gambassi et al., 2019).

Given these considerations, HRV serves as a valuable tool for monitoring training load, fatigue, recovery status, and performance optimization in athletes (Wang et al., 2025). We hypothesized that aerobic and anaerobic running would differentially influence ANS activity in male college athletes. Accordingly, the present study aimed to examine the acute effects of aerobic and anaerobic running on sympathetic and parasympathetic activity in college athletes, using both time- and frequency-domain HRV analyses.

The rationale for focusing specifically on aerobic versus anaerobic running lies in their distinct physiological and autonomic demands, which are well-established in the literature. Aerobic running primarily depends on sustained, oxygen-dependent energy metabolism and is generally associated with enhanced parasympathetic activation, whereas anaerobic running involves high-intensity, short-duration efforts that elicit increased sympathetic activation. By selecting these two contrasting exercise modalities, the present study aimed to investigate the acute autonomic responses and HRV changes within-subject design. We hypothesized that aerobic and anaerobic running would differentially affect sympathetic and parasympathetic activity in male college athletes. Accordingly, the study examined these effects using both time- and frequency-domain HRV analyses to provide a comprehensive assessment of ANS responses.

## Methods

### Subjects

Twenty-five healthy male college athletes as volunteers (mean ± SD age = 20.68 ± 1.651 years, weight = 74.08 ± 7.544 kg; height = 1.77 ± 0.041 m; sport age = 7.20 ± 2.27 years) participated in the study (**Table 1**). College athletes were students from the Faculty of Sports Sciences who were actively engaged in competitive sports across multiple disciplines, including football (n = 8), basketball (n = 5), handball (n = 2), taekwondo (n = 4), wrestling (n = 3), and volleyball (n = 3). In addition to completing at least 5 hours per week of practical sport-related classes as part of their curriculum, all participants regularly attended club training (≥3 sessions per week) and participated in at least one official competition per week, indicating a consistent level of training and competition exposure. Prior to the study, a G*Power analysis indicated that 28 participants would be required to achieve 80% power to detect a medium effect size. A total of 30 male college athletes were initially recruited. HRV recordings from 5 participants were unusable due to technical issues, resulting in a final sample of 25 participants who completed all exercise protocols.

**Table 1:** Physical characteristics of the participants (n=25).

Variables	Mean ± SD
Age (years)	20.68 ± 1.651
Weight (kg)	74.08 ± 7.544
Height (m)	1.77 ± 0.041
Sport age (years)	7.20 ± 2.27

### Procedures

All college athletes completed the exercise protocols in a fixed order, with the aerobic session performed first followed by the anaerobic session after a 24-hour interval. To minimize small and predictive effects, testing sessions were conducted under standardized conditions, including the same time of day, environmental settings, and pre-test instructions. Participants were experienced college athletes familiar with running and high-intensity exercise, which likely minimized learning effects. Baseline HRV measurements obtained prior to each session showed no significant differences, indicating that participants began both conditions under comparable autonomic states. Although the 24-hour interval appears sufficient for recovery, minor order or anticipatory effects cannot be entirely excluded and were considered in the study design. College athletes’ heart rates were continuously monitored using GPS devices. Based on literature indicating that the heart rate corresponding to a blood lactate level of 4 mmol/L typically ranges between 155 and 165 bpm, 160 bpm was used as the reference for the anaerobic threshold. Athletes were instructed to maintain aerobic running below 160 bpm and anaerobic running above 160 bpm, adjusting pace in real time according to GPS-monitored heart rate data. R-R interval data, heart rate, running speed, and duration were recorded at rest, post-run, and during both runs using the Polar V800 and H10 chest strap. The subjects abstained from smoking and coffee consumption for 12 h before the days of runs. Environmental conditions were maintained at 20.6 ± 1.08°C and 50% relative humidity. Aerobic and anaerobic running were taken for 2 days with a 24-hour interval.

### Aerobic running protocol

When athletes arrived at the test area for aerobic running, they were kept in a seated position for 2 minutes. After 2 minutes, a Polar watch and H10 chest strap were attached, and R-R recording was started. After resting for 10 minutes, the athletes completed an aerobic run of 9.57 ± 1.37 km/h (95% CI: 9.01–10.14) km/h with a mean heart rate of 152.09 ± 3.99 bpm (95% CI: 150.43–153.73) for 30.96 ± 1.05 minutes (95% CI: 29.91–32.01). Immediately after the run, the athlete was seated in a chair and allowed to recover for 10 minutes. The R-R recording was terminated after 10 minutes.

### Anaerobic running protocol

When athletes arrived at the test area for anaerobic running, they were kept in a seated position for 2 minutes. After 2 minutes, a Polar watch and H10 chest strap were fitted, and R-R recording was started. After resting for 10 minutes, the athletes completed an anaerobic run of 2.59 ± 0.15 minutes (95% CI: 2.43–2.76) min at 17.31 ± 3.52 km/h (95% CI: 15.93–18.68) km/h with a mean heart rate of 177.64 ± 2.60 bpm (95% CI: 174.76–180.99). Immediately after the run, the athlete was seated in a chair and allowed to recover for 10 minutes. R-R recording was terminated after 10 minutes.

### Data recording

R-R interval data were recorded using a POLAR Heart Rate Monitor (POLAR V800 NV, Finland) with a Polar H10 chest strap, at a sampling frequency of 1,000 Hz from the thoracic. R-R Interval Data Handling. Data were saved as R-R interval data files, with intervals in milliseconds (ms). For the Polar heart rate monitor raw, unfiltered R-R data were exported from the Polar Flow web service as a space delimited.txt file. Interval data were divided into the following sections: resting, during aerobic and anaerobic running, after running.

### HRV analysis

Kubios HRV is widely used in scientific research across disciplines such as cardiology, sports science, psychology, and stress research. Also, Kubios HRV software is employed in clinical studies to assess cardiovascular health and ANS function. Sports professionals utilize Kubios (Attar, 2024). The HRV data were analyzed by Kubios HRV scientific analysis software, version 4.1.1. This software processed the heart rate R-R intervals, extracting relevant parameters related to HRV in time domain, frequency domain, and non-linear analysis. For linear HRV analysis in the time domain, the RMSSD and pNN50 indices were included. The RMSSD and pNN50 indices reflect the PNS (Vanderlei et al., 2009). For non-linear analysis of HRV using geometric methods, the indices obtained from the Poincaré plane, SD1 and SD2 express the complexity of HRV. The SD1 index reflects the PNS, and the SD2 reflects the SNS and PNS (Vanderlei et al., 2009). The HF and LF index, both expressed in normalized units (nu), and the low-frequency/high-frequency (LF/HF) ratio were used as frequency domain analysis. The RMSSD and HF are well-established as sensitive indicators of PNS activity and have been used in previous studies (Marasingha-Arachchige et al., 2022; Dobbs et al., 2019; Kingsley and Figueroa, 2016). Frequency-domain HRV measures (LF, HF, LF/HF) were calculated to assess autonomic modulation. The LF component reflects both sympathetic and parasympathetic influences. While the LF/HF ratio has traditionally been used as an index of sympathovagal balance under stationary conditions (Marasingha-Arachchige et al., 2022; Kingsley and Figueroa, 2016), its interpretation during non-stationary conditions such as dynamic exercise is limited. Therefore, in the present study, these parameters were interpreted only as indicators of relative changes in autonomic modulation, rather than absolute measures of sympathetic–parasympathetic balance (Shaffer and Ginsberg, 2017). The HRV parameters used in this study (RMSSD, pNN50, HF, LF, LF/HF ratio, SD1, SD2, PNS index and SNS index) were based on those most examined in other studies (Grässler et al., 2021; Marasingha-Arachchige et al., 2022; Dobbs et al., 2019; Kingsley and Figueroa, 2016).

### Inclusion/exclusion criteria

Participants were 25 healthy male college athletes. Inclusion criteria included being free of chronic cardiovascular conditions and not using medications known to affect ANS function. Participants were instructed to abstain from smoking and caffeine for 12 hours prior to testing. While formal assessment of sleep quality was not conducted, all participants were physically active and accustomed to regular training, reducing the likelihood of confounding factors.

### Statistical analysis

All data were analyzed using SPSS 23.0 (IBM SPSS Statistics, Armonk, NY, USA). The normality of the variables was assessed using the Shapiro-Wilk test. Parametric tests were applied to normally distributed variables, whereas non-parametric tests were used for variables that did not meet the normality assumption. Within-subject effects over time were examined using repeated measures ANOVA for normally distributed variables and the Friedman test for non-normally distributed variables, with *post-hoc* pairwise comparisons performed using Bonferroni-adjusted t-tests or Wilcoxon signed-rank tests, respectively. Effect sizes were calculated as partial eta squared (η²) for ANOVA and Kendall’s W (ES) for Friedman tests, with values of 0.01, 0.06, and 0.14 for η² and 0.1, 0.3, and ≥0.5 for W indicating small, medium, and large effects, respectively. Comparisons between aerobic and anaerobic conditions were conducted using paired t-tests for normally distributed variables and Wilcoxon signed-rank tests for non-normally distributed variables. For the normally distributed variables SD1 and SD2, effect sizes for pairwise comparisons were further quantified using Cohen’s d for dependent samples: $d=\frac{\text{Mean Difference}}{\text{SD of the differences}}$ where the SD of the differences was derived from the standard error of the mean difference (SE) as: $SD_{\text{diff}}=SE\times \sqrt{n}$, Partial eta squared (η²_p_) for each paired comparison was also calculated from the t statistic using: $\eta_{p}^{2}=\frac{t^{2}}{t^{2}+df}$ where *t* is the t-value of the paired comparison and df=n−1. All figures illustrating pairwise comparisons, as well as differences between Aerobic Run (AeR) and Anaerobic Run (AnR), were generated using GraphPad Prism version 10.1.1 (GraphPad Software, California, USA). All data are presented as mean ± standard deviation (SD), and statistical significance was set at p< 0.05.

## Results

A statistically significant difference was found in HRV and ANS indices measurements at the rest, running, and recovery. This indicates a time-dependent change in HRV and ANS indices measurements for AeR (**Table 2**).

**Table 2:** Friedman test analysis of within-subject differences across rest, running, and recovery periods in the AeR.

HRV parameters	AeR	Mean ± SD	Mean rank	χ^2^	df	P	ES [Kendall’s W]
RMSSD (ms)	Resting	34.15 ± 16.88	2.96	42.560	2	.000^*^	.85
	Running	7.44 ± 12.02	1.12				
	Recovery	8.94 ± 4.57	1.92				
SD1 (ms)	Resting	26.17 ± 5.47	1.98	29.960	2	.000^*^	.60
	Running	33.72 ± 7.30	2.78				
	Recovery	20.96 ± 3.97	1.24				
SD2 (ms)	Resting	73.83 ± 5.47	2.02	29.960	2	.000^*^	.60
	Running	66.28 ± 7.30	1.22				
	Recovery	79.04 ± 3.97	2.76				
pNN50 (%)	Resting	10.72 ± 9.37	3.00	41.617	2	.000^*^	.83
	Running	0.39 ± 1.39	1.32				
	Recovery	0.66 ± 1.07	1.68				
LF (nu)	Resting	77.35 ± 12.02	1.80	8.720	2	.013^*^	.17
	Running	77.64 ± 10.36	1.72				
	Recovery	86.83 ± 4.89	2.48				
HF (nu)	Resting	22.63 ± 12.02	2.20	8.720	2	.013^*^	.17
	Running	22.30 ± 10.33	2.28				
	Recovery	13.15 ± 4.87	1.52				
LF**/**HF ratio	Resting	4.53 ± 2.54	1.80	8.720	2	.013^*^	.17
	Running	4.69 ± 3.26	1.72				
	Recovery	7.48 ± 2.68	2.48				
ANS indices parameters							
PNS index	Resting	-1.46 ± 0.85	3.00	50.000	2	.000^*^	1.0
	Running	-4.19 ± 0.41	1.00				
	Recovery	-3.16 ± 0.43	2.00				
SNS index	Resting	1.83 ± 1.26	3.00	50.000	2	.000^*^	1.0
	Running	12.62 ± 3.07	1.00				
	Recovery	6.40 ± 1.97	2.00				

SD, standard deviation; HRV, heart rate variability; RMSSD, root mean square of successive differences between adjacent normal R-R intervals; pNN50, Percentage of adjacent RR intervals with duration difference greater than 50 ms; HF, high-frequency; LF, low-frequency; LF/HF ratio, high-frequency/low-frequency ratio; ANS, Autonomic nervous system; PNS, Parasympathetic nervous system; SNS, Sympathetic nervous system.*p<.05.

A statistically significant difference was found in HRV and ANS indices measurements at the rest, running, and recovery. This indicates a time-dependent change in HRV and ANS indices measurements for AnR (**Table 3**).

**Table 3:** Friedman test analysis of within-subject differences across rest, running, and recovery periods in the AnR.

HRV parameters	AnR	Mean ± SD	Mean rank	χ^2^	df	P	ES [Kendall’s W]
RMSSD (ms)	Rest	35.36 ± 18.30	2.96	34.880	2	.000^*^	.70
	Running	8.88 ± 15.11	1.60				
	Recovery	5.52 ± 2.61	1.44				
pNN50 (%)	Rest	13.79 ± 11.46	2.96	44.718	2	.000^*^	.90
	Running	0.75 ± 2.75	1.60				
	Recovery	0.06 ± 0.21	1.44				
LF (nu)	Rest	79.27 ± 11.51	1.96	30.480	2	.000^*^	.61
	Running	49.16 ± 20.96	1.24				
	Recovery	87.00 ± 6.96	2.80				
HF (nu)	Rest	20.49 ± 11.46	2.04	30.480	2	.000^*^	.61
	Running	50.72 ± 20.90	2.76				
	Recovery	6.95 ± 5.43	1.20				
LF**/**HF ratio	Rest	5.60 ± 4.31	1.96	30.480	2	.000^*^	.61
	Running	1.59 ± 1.85	1.24				
	Recovery	8.80 ± 4.49	2.80				
ANS indices parameters							
PNS index	Rest	-1.37 ± 1.13	3.00	48.080	2	.000^*^	.96
	Running	-4.32 ± 0.61	1.04				
	Recovery	-3.35 ± 0.37	1.96				
SNS index	Rest	2.07 ± 2.13	1.00	44.720	2	.000^*^	.89
	Running	16.60 ± 5.58	2.88				
	Recovery	9.62 ± 2.95	2.12				

*p<.05.

**Figure 1:**
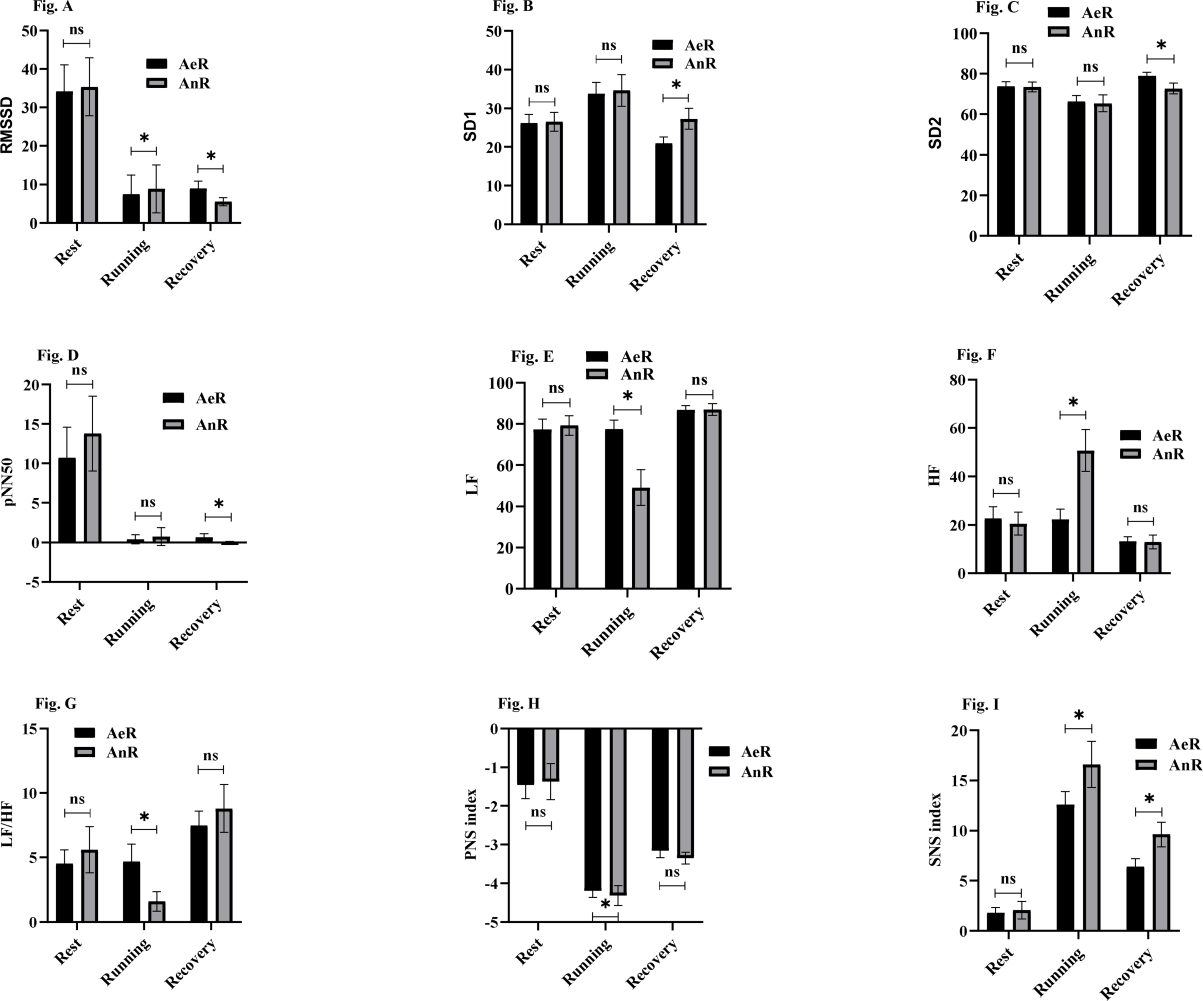
Between across rest, running, and recovery periods in AeR and AnR. #RMSSD **(A)**, SD1 **(B)**, SD2 **(C**, pNN50 **(D)**, LF **(E)**, HF **(F)**, LF/HF **(G)**, PNS index **(H)**, and SNS index **(I)** across rest, running, and recovery periods in the AeR and AnR groups. *p<.05.

No significant differences were found in RMSSD, SD1, SD2, pNN50, LF, HF, LF/HF, SNS index, and PNS index for rest. Running, statistically significant differences were found between AeR and AnR in terms of RMSSD, LF, HF, LF/HF, PNS index and SNS index. RMSSD, HF, PNS, and SNS values were lower during AeR compared to AnR, whereas LF and LF/HF values were higher (**Figure 1**).

During anaerobic running, HF increased while LF and LF/HF decreased (**Figures 1–G**). Considering that interpretation of these parameters under non-stationary conditions, such as dynamic exercise, is limited, these changes are interpreted as relative shifts in autonomic modulation rather than absolute measures of sympathetic or parasympathetic activity. Specifically, the pattern may suggest a relative enhancement of parasympathetic influence or reduced sympathetic modulation during anaerobic exercise (**Figure 1**).

During recovery, SD1 and SNS index were lower after aerobic running compared to anaerobic running, whereas RMSSD, SD2, and pNN50 were relatively higher (**Figures 1A–D, I**), indicating relatively reduced short-term autonomic modulation during aerobic recovery and greater parasympathetic activity during anaerobic recovery (**Figure 1**).

Mauchly’s Test of Sphericity was significant for both SD1 and SD2 (p<.05), indicating that the assumption of sphericity was violated; therefore, the Greenhouse-Geisser correction was applied. The results show that time had a statistically significant effect on both SD1 and SD2 across the measurement points (Rest, Anaerobic Run, Recovery), with a very large effect size (η²* = .398), indicating that approximately 39.8% of the variance in each variable is explained by time. These findings suggest that SD1 and SD2 changed substantially across the three measurement points (**Table 4**).

**Table 4:** Within-subject effects across rest, running, and recovery periods: a repeated measures ANOVA.

HRV parameters	AnR	Mean ± SD	Type III Sum of Squares	df	Mean Square	F	P	η²p
SD1 (ms)	Rest	26.51 ± 5.92	2781.81	1.60	1744.26	31.737	.001^*^	.398
	Running	34.58 ± 9.91						
	Recovery	27.26 ± 6.53						
Error			4207.28	76.55	54.959			
SD2 (ms)	Rest	73.49 ± 5.92	2781.07	1.60	1743.83	31.725	.001^*^	.398
	Running	65.42 ± 9.91						
	Recovery	72.74 ± 6.53						
Error			4207.73	76.55	76.246			

SD1, SD of the Poincaré plot width; and SD2, SD of the length of the Poincaré plot.

**Figure 2:**
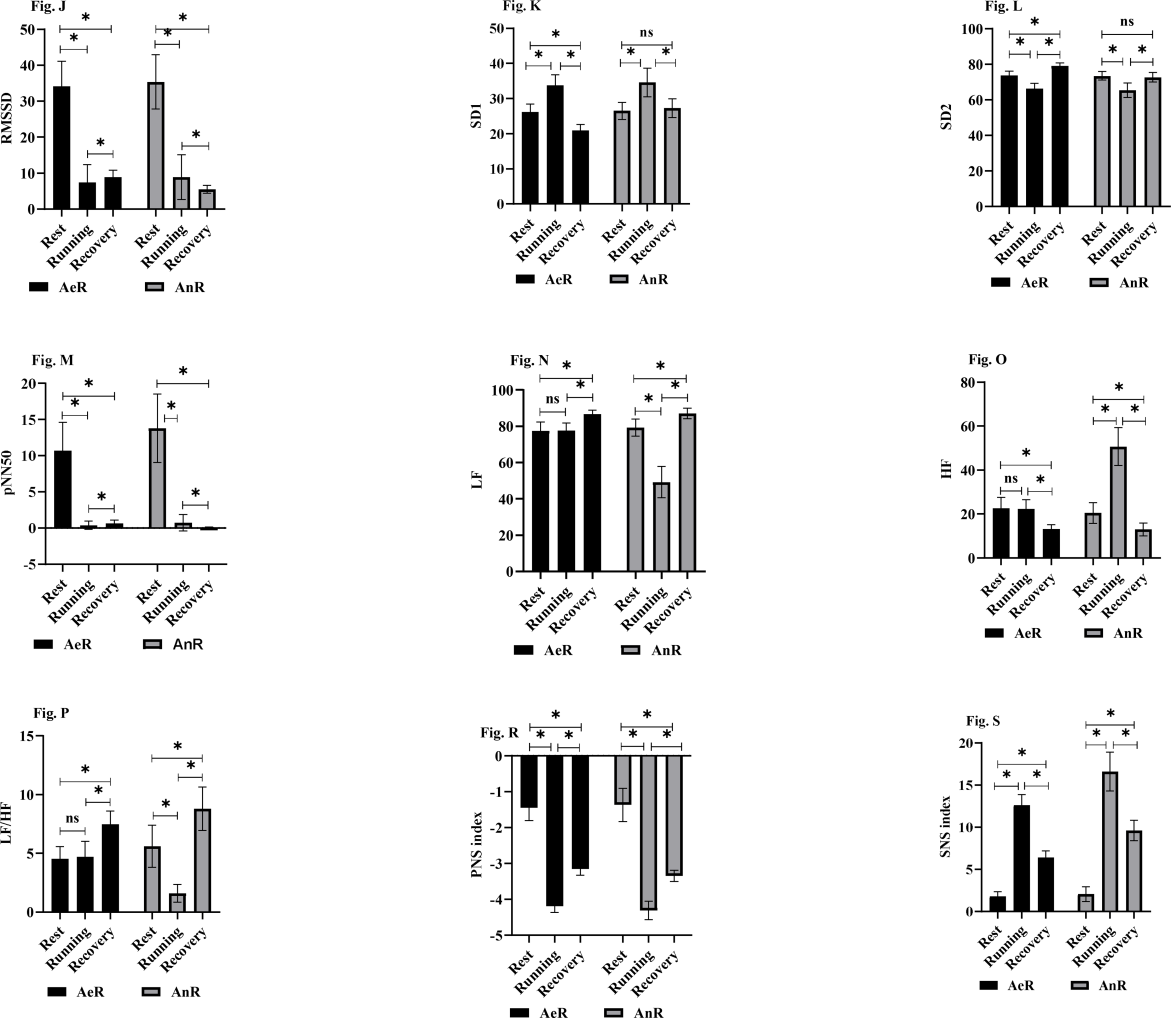
Within-subject differences across rest, running, and recovery periods in AeR and AnR. #Within-subject differences across rest, running, and recovery periods in RMSSD **(J)**, SD1 **(K)**, SD2 **(L)**, pNN50 **(M)**, LF **(N)**, HF **(O)**, LF/HF **(P)**, PNS index **(R)**, and SNS index **(S)** in the AeR and AnR groups. *p<.05.

In terms of comparisons across measurement points, both SD1 and SD2 showed significant changes, with substantial increases from Rest to Running (AnR; SD1: p<.001, d = 1.03, η² = 0.52; SD2: p<.001, d = 1.03, η² = 0.53) and from Running to Recovery (SD1: p<.001, d = 1.40, η² = 0.67; SD2: p<.001, d = 1.40, η² = 0.67), while changes from Rest to Recovery were not statistically significant (SD1: p = .064; SD2: p = .065) despite moderate effect sizes (d ≈ 0.48, η² ≈ 0.19), highlighting notable changes particularly during the AnR phase (**Figures 2K, L**).

As shown in **Figure 2**, in terms of AeR, a significant decrease in parasympathetic activity parameters (RMSSD, pNN50, PNS index) is observed during aerobic running. This indicates that aerobic running exerts parasympathetic pressure on the cardioautonomic system and that sympathetic activity (SD1, SNS index) increases slightly. The absence of significant changes in LF, HF, and the LF/HF ratio suggests that the overall sympathetic-parasympathetic balance is largely maintained, except for a brief disruption. During the recovery period, while a recovery in parasympathetic parameters is observed, some indicators (HF, SD1) remain below baseline levels. This indicates that recovery after aerobic running has not fully begun and that there is a short-term shift in cardioautonomic balance. At the same time, the increase in sympathetic activity parameters (SD2, LF, LF/HF, SNS index) can be considered a reflection of the short-term stimulating effect of aerobic running. These results demonstrate that aerobic running transiently affects heart rate and beat-to-beat variability via parasympathetic inhibition, while the sympathetic system responds with mild compensatory activation.

During anaerobic running (AnR), RMSSD, pNN50, SD2, and PNS index decreased, while SD1 and SNS index increased. HF increased and LF/HF decreased; although this may superficially suggest enhanced parasympathetic activity, LF and HF changes can reflect spectral artifacts during high-intensity, non-stationary exercise (Billman, 2013). Overall, SD1 and SNS index indicate sympathetic dominance during anaerobic running. During recovery, short-term parasympathetic indicators (RMSSD, SD1, pNN50, HF) and SNS showed a decrease, while long-term and frequency-domain parameters (SD2, LF, LF/HF, PNS) showed partial recovery, indicating a parameter-specific, persistent sympathetic effect post-exercise.

In terms of ANS Indices, in both groups, running shifts the autonomic balance from parasympathetic to sympathetic by decreasing the PNS index and increasing the SNS index; this effect varies depending on the intensity of the run. AeR slightly suppresses parasympathetic activity and briefly increases sympathetic activity; with post-run recovery, the PNS index partially increases while the SNS index approaches normal. This indicates that aerobic running has a limited and temporary effect and that the parasympathetic system is briefly suppressed. AnR, on the other hand, exerts parasympathetic suppression more strongly and significantly increases sympathetic activation; post-anaerobic run recovery is limited, the PNS index remains below baseline, while the SNS index remains high, revealing a shift in the autonomic balance towards a sympathetically oriented profile.

## Discussion

The present study demonstrates that both aerobic (AeR) and anaerobic running (AnR) induce significant alterations in cardiac autonomic regulation, characterized by a shift toward sympathetic predominance and parasympathetic withdrawal. However, beyond this general pattern, our findings indicate that the magnitude and recovery profile of these responses are strongly dependent on exercise intensity and are underpinned by distinct physiological mechanisms.

From a mechanistic perspective, the autonomic adjustments observed during AeR can largely be attributed to central command and mild activation of muscle mechanoreceptors, which primarily drive vagal withdrawal at exercise onset. In this condition, sympathetic activation remains relatively limited, likely due to lower catecholamine release and minimal arterial baroreflex resetting, resulting in transient reductions in vagally mediated HRV indices (e.g., RMSSD, pNN50, PNS index) and a rapid post-exercise restoration of autonomic balance. In contrast, the more pronounced autonomic perturbation observed during AnR reflects the combined effects of central command, enhanced muscle metaboreflex activation, and substantial increases in circulating catecholamines (epinephrine and norepinephrine). The accumulation of metabolic by-products such as lactate and H^+^ ions stimulate group III–IV afferents, further augmenting sympathetic outflow. Simultaneously, baroreceptors are reset to operate at higher arterial pressure levels, sustaining sympathetic activation despite elevated blood pressure. These mechanisms provide a physiological basis for the greater suppression of parasympathetic activity and the stronger sympathetic dominance observed in HRV parameters during high-intensity running.

The recovery phase further highlights these intensity-dependent differences. While AeR is associated with rapid vagal reactivation due to the quick withdrawal of central command and limited metabolic disturbance, AnR results in delayed recovery, likely driven by sustained metaboreflex activation, slower metabolite clearance, and prolonged catecholaminergic effects. Although the LF/HF ratio is frequently used to assess sympathetic and parasympathetic activity during exercise, recent evidence highlights its limitations. In particular, during incremental exercise, the LF/HF ratio often fails to accurately reflect changes in sympathetic activity. In a study by Tanoue et al. (2022), while LF/HF did not show a significant increase with rising exercise intensity, the heart rate/LF ratio exhibited a nonlinear increase that paralleled changes in norepinephrine and blood lactate levels. Notably, heart rate/LF values were positively correlated with norepinephrine, blood lactate, and carbon dioxide production from rest through the stages of exercise. These findings suggest that the LF/HF ratio alone may be insufficient to capture SNS activity, whereas the heart rate/LF ratio represents a more reliable HRV-based index for evaluating sympathetic activity and metabolic responses during incremental exercise.

In a study, the indicator of PNS activity decreased dramatically when the subjects exercised compared with rest and continued to decrease until the intensity reached 60% ventilatory threshold. The indicator of SNS activity remained unchanged up to 100% ventilatory threshold, whereas it increased abruptly at 110% ventilatory threshold (Yamamoto et al., 1991). In a previous study by Shin et al. (1995), the effects of long-term physical training on autonomic function in athletes and the response of the ANS to dynamic exercise were investigated in nonathletes and athletes with power spectral analysis of HRV. In athletes and nonathletes, LF and HF powers gradually decreased with exercise. As recovery progressed, they continued to increase gradually but remained below resting level. During rest and postexercise, HF power in athletes was significantly higher than that in non-athletes. Also, the recovery of HR and HF power during early recovery was more rapid in athletes than in nonathletes. Both groups showed an attenuation of LF and HF powers during dynamic exercise. In a study, exercise was performed using either upper body (arm ergometer), lower body (cycle) or whole body (treadmill) modes.

Compared to whole or lower body exercise, upper body exercise resulted in significantly greater measures of HRV particularly those within the very low (0–0.04 Hz) and low (0.04–0.15 Hz) frequency bands (Leicht et al., 2008). The HRV at the end of exercise and recovery was assessed with Fast Fourier Transform as well. The results showed that LF power and HF power during the recovery period were affected by exercise intensity, recovery time and their interaction (Martinmäki and Rusko, 2008). In a previous study, the LF and HF components decreased significantly at the onset of exercise. However, with increasing exercise intensity, the HF component expressed as normalized units (n.u.) (reflecting parasympathetic modulation) increased significantly, whereas the LF component (n.u.) and LF/HF ratio (both reflecting sympathetic modulation) decreased significantly (Pichon et al., 2004).

In a study shown that resistance training groups had significantly higher RMSSD, SD1, and SD2 values compared to the control group, reflecting greater parasympathetic activity, which is that long-term resistance training enhances parasympathetic modulation (Barbosa et al., 2025). There was a significant increase in the difference of the triangular index, SD1, SD2, and RR intervals in the functional training exposed periodized for 12 weeks and 23.00 ± 2.51 years women as compared to the control group. Functional training has been reported to have a beneficial effect on autonomic modulation, characterized by increased parasympathetic activity and global variability (de Rezende Barbosa et al., 2016).

**In conclusion,** at baseline, a relatively balanced autonomic state with parasympathetic predominance was observed prior to both aerobic and anaerobic running, which enhances the interpretability of the findings.

During exercise, SNS activity increased in both conditions, with a more pronounced response observed during anaerobic running compared to aerobic running. This finding indicates that ANS responses are strongly influenced by exercise intensity.

During the 10-minute post-exercise recovery period, parasympathetic activity tended to return toward pre-exercise levels, whereas sympathetic activity remained relatively elevated. This suggests that autonomic recovery, particularly following anaerobic exercise, may require a longer duration.

The present findings demonstrate that both aerobic and anaerobic exercise induce short-term, measurable, and parameter-specific alterations in HRV and ANS indices. From a physiological perspective, the greater sympathetic activation observed during anaerobic exercise reflects increased central command, catecholamine release, and baroreflex adjustments associated with higher exercise intensity.

Importantly, the interpretation of frequency-domain HRV parameters, particularly HF, should be approached with caution during high-intensity, non-stationary exercise conditions, as these measures may be influenced by respiratory rate, mechanical ventilation, and signal non-stationarity rather than pure vagal modulation. In this context, the separate evaluation of parasympathetic and sympathetic indices is essential for accurate interpretation of autonomic responses in both experimental and clinical settings. Furthermore, the findings support that anaerobic exercise exerts more pronounced effects on HRV parameters and that autonomic balance is highly sensitive to exercise intensity.

## Limitation

Several limitations should be acknowledged when interpreting the present findings. Participants were healthy male college athletes screened for cardiovascular disease and medications affecting autonomic function; however, formal assessment of sleep quality, psychosocial status, and other subclinical factors was not conducted. Acute and chronic psychosocial stress may influence HRV and autonomic responses, yet the standardized assessment and control of these variables were not feasible due to the field-based and acute exercise-oriented design of the study. Therefore, these factors may have affected autonomic responses and should be considered when interpreting the findings.

The study employed a within-subject design with a fixed exercise order (aerobic session first, anaerobic session 24 h later), which may have introduced order effects such as residual fatigue or minor familiarization, despite baseline HRV measures indicating comparable autonomic states before each session. Furthermore, the aerobic session was performed first to minimize the possibility of acute fatigue affecting subsequent measurements. Baseline HRV measurements before the sessions showed similar autonomic states; however, the fixed-order design still presents a methodological limitation because minor effects related to residual fatigue, order effects, and familiarity cannot be completely excluded. Mixed/balanced designs may represent a suitable alternative for reducing rank effects, yet a fixed-rank design was preferred in this study to ensure standardization in HRV measurements and reduce inter-individual variability. Participants were physically active and experienced with high-intensity exercise, which likely minimized learning effects and potential confounding related to unfamiliarity.

Additional methodological limitations should also be acknowledged. Exercise intensities were not individually prescribed based on VO_2_max, lactate threshold, or other objective physiological markers, limiting precise classification of aerobic and anaerobic effort. In addition, the absence of detailed training-load quantification and the heterogeneous nature of the sample, which included athletes from multiple sport disciplines, may have contributed to variability in HRV responses. HRV was measured during non-stationary high-intensity exercise; therefore, certain interpretations, particularly the use of the LF/HF ratio as an indicator of sympatho-vagal balance, should be interpreted cautiously, as this approach may reflect outdated assumptions.

HRV was assessed using both linear and nonlinear methods. Time- and frequency-domain indices were complemented with nonlinear Poincaré plot-derived measures (SD1 and SD2). However, more advanced phase-domain nonlinear metrics, such as pointwise correlation dimension (PD2) and entropy-based measures, were not included, which is acknowledged as a methodological limitation.

Despite these limitations, several methodological precautions were implemented, including standardized testing conditions, baseline HRV control, and the inclusion of exercise-experienced participants, which may have helped reduce potential confounding effects. Future studies could strengthen internal validity through randomized or counterbalanced protocols, individualized exercise-intensity prescriptions, objective physiological markers (e.g., VO_2_max or lactate thresholds), familiarization sessions, and longer recovery intervals. In addition, incorporating high-frequency physiological and psychological monitoring within more comprehensive individualized study designs may provide deeper insights into autonomic regulation during different exercise modalities.

## Data Availability

The raw data supporting the conclusions of this article will be made available by the authors, without undue reservation.
